# Potential for subsets of wt-NPM1 primary AML blasts to respond to retinoic acid treatment

**DOI:** 10.18632/oncotarget.23642

**Published:** 2017-12-23

**Authors:** Rodica P. Bunaciu, Robert J. MacDonald, Feng Gao, Lynn M. Johnson, Jeffrey D. Varner, Xin Wang, Sarah Nataraj, Monica L. Guzman, Andrew Yen

**Affiliations:** ^1^ Department of Biomedical Sciences, Cornell University, Ithaca, NY, USA; ^2^ Division of Hematology and Medical Oncology, Department of Medicine, Weill Cornell Medical College, New York, NY, USA; ^3^ Cornell Statistical Unit, Cornell University, Ithaca, NY, USA; ^4^ Robert Frederick Smith School of Chemical and Biomolecular Engineering, Cornell University, Ithaca, NY, USA; ^5^ Department of Biomedical Sciences, City University of Hong Kong, Hong Kong, China

**Keywords:** retinoic acid, c-Raf, c-Cbl, AML, precision oncology

## Abstract

Acute myeloid leukemia (AML) has high mortality rates, perhaps reflecting a lack of understanding of the molecular diversity in various subtypes and a lack of known actionable targets. There are currently 12 open clinical trials for AML using combination therapeutic modalities including all-*trans* retinoic acid (RA). Mutant nucleophosmin-1, proposed as a possible marker for RA response, is the criterion for recruiting patients in three active RA phase 3 clinical trials. We tested the ability of RA alone or in combination with either bosutinib (B) or 6-formylindolo(3,2-b) carbazole (F) to induce conversion of 12 *de novo* AML samples toward a more differentiated phenotype. We assessed levels of expression of cell surface markers associated with differentiation, aldehyde dehydrogenase activity, and glucose uptake activity. Colony formation capacity was reduced with the combined treatment of RA and B or F, and correlated with modulation of a c-Cbl/Lyn/c-Raf-centered signalsome. Combination treatment was in most cases more effective than RA alone. Based on their responses to the treatments, some primary leukemic samples cluster closer to HL-60 cells than to other primary samples, suggesting that they may represent a hitherto undefined AML subtype that is potentially responsive to RA in a combination differentiation therapy.

## INTRODUCTION

Acute myeloid leukemia (AML) is the most frequently occurring adult leukemia (comprising about 30% of new leukemias). It is also is the most deadly (causing 43% of leukemia deaths yearly), with a five-year survival rate below 27%, whereas other leukemias have a survival rate over 66% [1, 2]. Since the 1970s, the AML five-year survival rate has increased from 6.3% to 26.9% (in 2013). However, incidence rates also increased [2]. Significantly, in female adolescents, cancer incidence over the previous 38 years increased by 61%. For them, AML incidence- is surpassed by only Non-Hodgkin lymphoma and thyroid cancer [3]. In the USA, the estimated 2017 numbers for AML are 21,380 new cases and 10,590 new deaths [2]. There is thus great need for new insights that motivate better treatment modalities.

The poor five-year survival rate is due to very high heterogeneity in etiology, incomplete understanding of molecular signature seminal to different subtypes, and lack of actionable targets and precision therapies. Many AML cases present recurrent genetic abnormalities (Table 1) [4–22]. In any given AML patient, multiple genetic abnormalities are frequently present. The number of these genetic abnormalities increases with relapse [23]. The high genetic/epigenetic heterogeneity is associated with widespread perturbations of growth and differentiation regulatory signaling networks. There are currently 12 open clinical trials for non-acute promyelocytic leukemia (non-APL) AML using combination therapeutic modalities including retinoic acid (RA), a very successful differentiating agent in the case of APL. Those include NCT01237808, NCT00893399, NCT03031249, phase-3 clinical trials for adult patients with AML and nucleophosmin (NPM1) mutation. The clinical trials illustrate the current unmet medical need of finding oncotargets such as NPM1 and agents such as RA that can mitigate the transformed molecular network and improve survival.

**Table 1:** Most frequent genetic abnormalities in AML

Most frequent karyotypic aberrations	Notes
t(8;21)(q22;q22.1); RUNX1-RUNX1T1	7% of adults with AML and most children with AML
inv(16)(p13.1q22) or t(16;16)(p13.1;q22); CBFB-MYH11	5% of adults with *de novo* AML
PML-RARA, t(9;11)(p21.3;q23.3); MLLT3-KMT2A	6% of young adults with *de novo* AML and up to 12% of children with AML
t(6;9)(p23;q34.1); DEK-NUP214	1% of adults and approximately 10% of children with *de novo* AML
inv(3)(q21.3q26.2) or t(3;3)(q21.3;q26.2); GATA2,MECOM	1% of AML cases
t(1;22)(p13.3;q13.3); RBM15-MKL1	less than 0.5% of AML
BCR-ABL1	2% of AML and 38% of mixed- phenotype acute leukemia and presents poor prognosis
Chromosome 5 genetic defects such as monosomy 5 or del(5q)	frequently involved in myelodysplastic syndrome (MDS) and AML with MDS-related features
**Frequently Mutated Genes in AML**	**Notes**
NPM1	25%-50% *de novo* AML but not secondary AML. Usually mut-NPM1 confers better prognosis and increased response to chemotherapy.
CEBPA	CCAAT/enhancer binding protein alpha biallelic mutations confer better prognosis
RUNX1	3–33% of people with MDS and AML
FLT3	FLT3-internal tandem duplications confer adverse prognosis. 10%-30% of patients with cytogenetically normal AML
cKIT	6% of *de novo* AML
IDH1/2	15% *de novo* AML

Given the high heterogeneity of AML pathology, improving AML patient survival rates depends on characterizing the networks that link genetic defects to molecular signaling and molecular signaling to cellular functional outcomes. To gain mechanistic insights that reveal diagnostic markers and targets of therapeutic intervention, a longstanding approach has been analysis of *in vitro* models, which provide an experimentally tractable and simplified context for pathway analysis. We previously characterized an ensemble of proteins that form a putative complex that generates signaling seminal to RA-induced differentiation of a patient-derived non-APL AML cell model, the HL-60 cell line. The signaling complex embodies molecules historically found to drive mitogenesis upon stimulation by growth factors, but drive differentiation upon stimulation by RA. Prominent members include E3 ubiquitin-protein ligase CBL (c-Cbl) [24–26], Raf-1 proto-oncogene, serine/threonine kinase (c-Raf) [24, 27–29], aryl hydrocarbon receptor (AhR) [24, 30], LYN proto-oncogene, Src family tyrosine kinase (Lyn) [24, 28], and interferon regulatory factor 1 (IRF1) [31]. We posited that there is a signalsome comprised of those molecules [24, 32–35] with a notable kinase module [28, 29, 31, 32, 34, 35]. These contribute to a molecular signature betraying pathway activation in response to RA. Cotreatment with either AhR agonists [24, 30, 32, 36] or Src family kinase (SFK) inhibitors [24, 28, 30, 35, 36] enhances these and other potentially collaborating signals. Strikingly, a core of signaling molecules are similarly regulated by both the AhR agonists and SFK inhibitors [24, 28, 32, 35, 37]. Consistent with inferences from the model, the AhR antagonist StemRegenin has been reported to promote expansion of human hematopoietic stem cells [38, 39] and a multi-kinase tyrosine kinase inhibitor, midostaurin, has been FDA approved for treatment of FLT3 positive AML [40]. Hence AhR agonists and SFK inhibitors may target regulatory molecules in a common network embodied by this putative signalsome.

In the present studies, we show first that in mut-NPM1 but not wt-NPM1 patients c-Cbl is a marker for discriminating AML response to cytotoxic chemotherapy. We then look at the correlation of NPM1 and markers of differentiation status. Using this data to select a limited number of samples that embodied a diversity of traits, we tested their response to RA or RA plus a small molecule targeting AhR, measuring their colony forming/growth capacity in methylcellulose. The AhR agonist was 6-formylindolo(3,2-b) carbazole, i.e. FICZ (F). A SFK inhibitor bosutinib (B) was also tried but it was not as consistently effective in reducing the colony forming capacity. We then characterized the signaling signature associated with response to this combination of RA plus F to reveal a c-Cbl related signaling telltale predictive of response. Finally we determined if there was an AML subtype that embodied this signature.

From these studies, we report four main conclusions. 1. c-Cbl used as a prognostic indicator with NPM1 results in better disease-free survival (DFS) stratification than NPM1 alone; in particular, AML with mut-NPM1 and low c-Cbl expression performed better than high c-Cbl. 2. Low colony formation capacity occurred in samples where RA could regulate signaling, specifically c-Cbl, Lyn and c-Raf/phospho c-Raf. 3. RA-induced responses could be enhanced by combining RA with F or B. 4. The widely used patient–derived RA-responsive HL-60 AML cell line bore fidelity to a hitherto uncharacterized AML subtype with wt-NPM1, low CD34 where c-Cbl, Lyn and c-Raf/phospho c-Raf could be regulated by RA. We ergo put forth the conjecture that there is an AML subtype that can respond to RA in combination with other agents, and it is captured *in vitro* by the HL-60 cell line.

## RESULTS

### c-Cbl expression is a prognostic biomarker for further stratification of patients with mut-NPM1 AML

**Figure 1:**
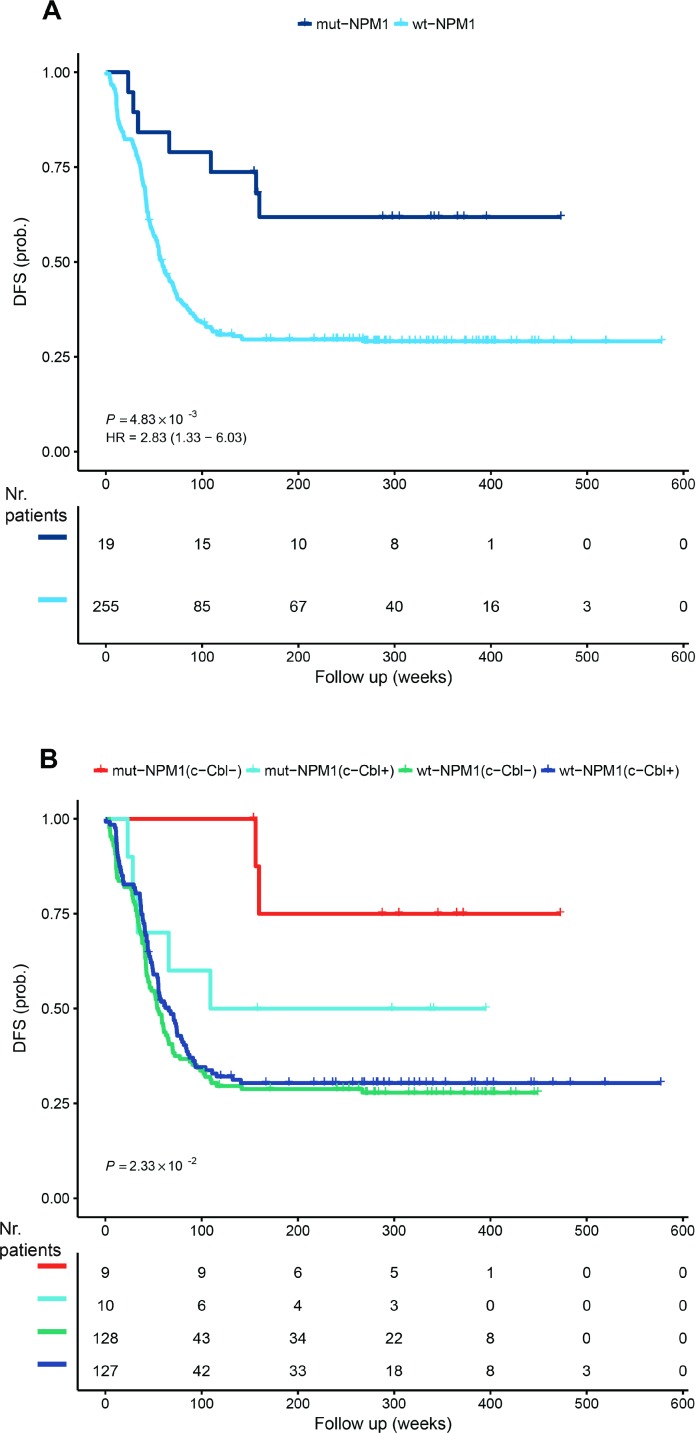
Disease-free survival The TARGET RNA-Seq data set was analyzed for disease-free survival dependence on NPM1 (**A**) and NPM1 and c-Cbl (**B**) dependence. c-Cbl- and c-Cbl+ represents low and respective high c-Cbl mRNA expression. The analysis was performed using the R package ‘survival’ (https://cran.r-project.org/package=survival) and the *p* value was calculated from the log-rank test. HR represents hazard rates. The number of patients during the follow up weeks is included stratified for c-Cbl and NPM1.

NPM1 is a known stratification marker for AML. The 2016 WHO classification includes a subtype of AML with recurrent genetic abnormalities consisting of mutations of *NPM1* [17]. The prognosis is considered to be better for this subtype [41, 42]. In the TARGET NIH AML database, we calculated the average overall survival for mut-NPM1 to be 1781.75 days and for wt-NPM1 to be 1406.3 days. Moreover, the DFS curves also confirm reported data [41, 42] that mut-NPM1 patients have a more favorable outcome (Figure 1A). The existence of NPM1 gene mutations is a criterion for recruiting patients in three active RA phase 3 clinical trials (NCT01237808, NCT00893399 and NCT03031249).

We previously reported a signalsome that generates signaling seminal to RA-induced differentiation in a well-studied patient derived non-APL AML cell line, HL-60, that responds to RA and differentiates with cell cycle arrest. One of the members of the signalsome is the c-Cbl adaptor/E3-ligase, which has been implicated in myelodysplastic syndromes [43] and AML [44–46]. We have previously shown that c-Cbl in the signalsome is a nexus for partners, and its expression regulates differentiation [24–26, 31, 32, 35–37, 47]. Therefore, we asked if c-Cbl expression at presentation can substratify the NPM1 stratification of the survival curve. As presented in Figure 1B, the most favorable outcome occurs with c-Cbl low expression and mut-NPM1, whereas high c-Cbl expression and mut-NPM1 approaches the wt-NPM1 DFS curve.

The data presented in Figure 1B accounts for just c-Cbl mRNA expression. However, it is known that c-Cbl and SFKs, for example, interact. Furthermore, other kinase modules are known to integrate signals from c-Cbl or from SFKs. Therefore, we hypothesized that prediction of RA responsiveness in non-APL AML blasts predicated on NPM1 status can be further refined by the signalsome response. Specifically, we hypothesized that the subset of wt-NPM1 cases that exhibited signalsome modulation would have a better response to RA than the wt-NPM1 cases that did not exhibit signalsome modulation. Cell surface proteins are routinely used as markers of differentiation. Therefore, we tested the clustering of treated and untreated *de novo* non-APL AML blasts, based on expression of surface markers.

### Some non-APL AML wt-NPM1 blasts cluster closer to mut-NPM1

**Figure 2:**
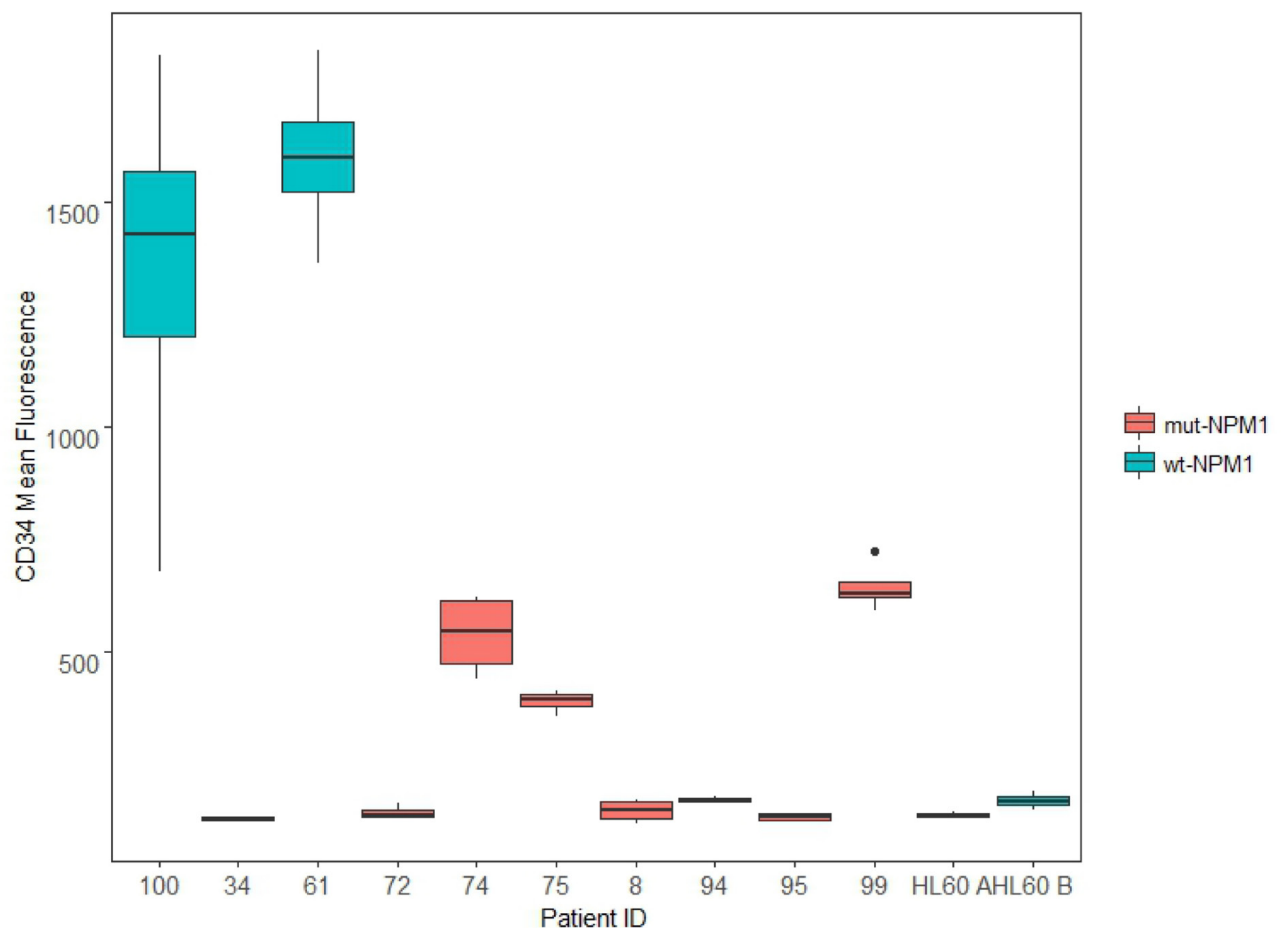
CD34 expression *De novo* patient samples and HL-60 samples, untreated and treated for 72 h (RA, FRA, BRA), were analyzed for CD34 expression using CD34 APC antibody. Mean expression of the singlet population for each sample is compared using the ggplot2 package in R. For each sample, the wider the boxplot, the greater difference in CD34 expression among C, RA, FRA, BRA. AML 100, AML 61, AML 94 and HL-60 cell line are wt-NPM1.

12 *de novo* AML patient samples were assessed for their NPM1 status. Of the samples, 5 were wt-NPM1: AML 37; AML 61; AML 76; AML 94; AML 100; and 7 were mut-NPM1: AML 8; AML 34; AML 72; AML 74; AML 75; AML 95; and AML 99. HL-60 was also assessed and determined to be wt-NPM1. About 70% of the wt-NPM1 exhibit high expression of CD34 [10]. We next assessed expression of this marker by flow cytometry in several samples. For each sample, 4 cases were considered. These were untreated, RA treated, and because AhR and SFKs are c-Cbl linked, RA plus F, an AhR ligand, and RA plus B, an SFK ligand (respectively designated RA-, FRA-, BRA-treated). To see if a relationship between NPM1 and CD34 emerged, we graphed the results using a boxplot, where all 4 cases (C, RA, FRA, and BRA) for each sample form a box (Figure 2). AML 100, AML 61, AML 94 and HL-60 cell line are wt-NPM1.

Two wt-NPM1 samples (AML 100 and AML 61) have very high CD34 expression, and the response to treatment is greater in AML 100 than in AML 61. One wt-NPM1 sample (AML 94) has very low CD34 expression, lower than that of several mutants (AML 74, AML 75, AML 99). HL-60 cells, which are wt-NPM1 cells, at two different passages (A and B) also have low CD34 expression. AML 94 and AML 100 exhibit diverse total CD34 expression. It is thus evident that NPM1 status per se by itself is not directing CD34 expression. This is consistent with the notion put forth above that NPM1 status reflects a heterogeneous population where NPM1 should be analyzed with other combinations of markers, e.g. c-Cbl.

**Figure 3:**
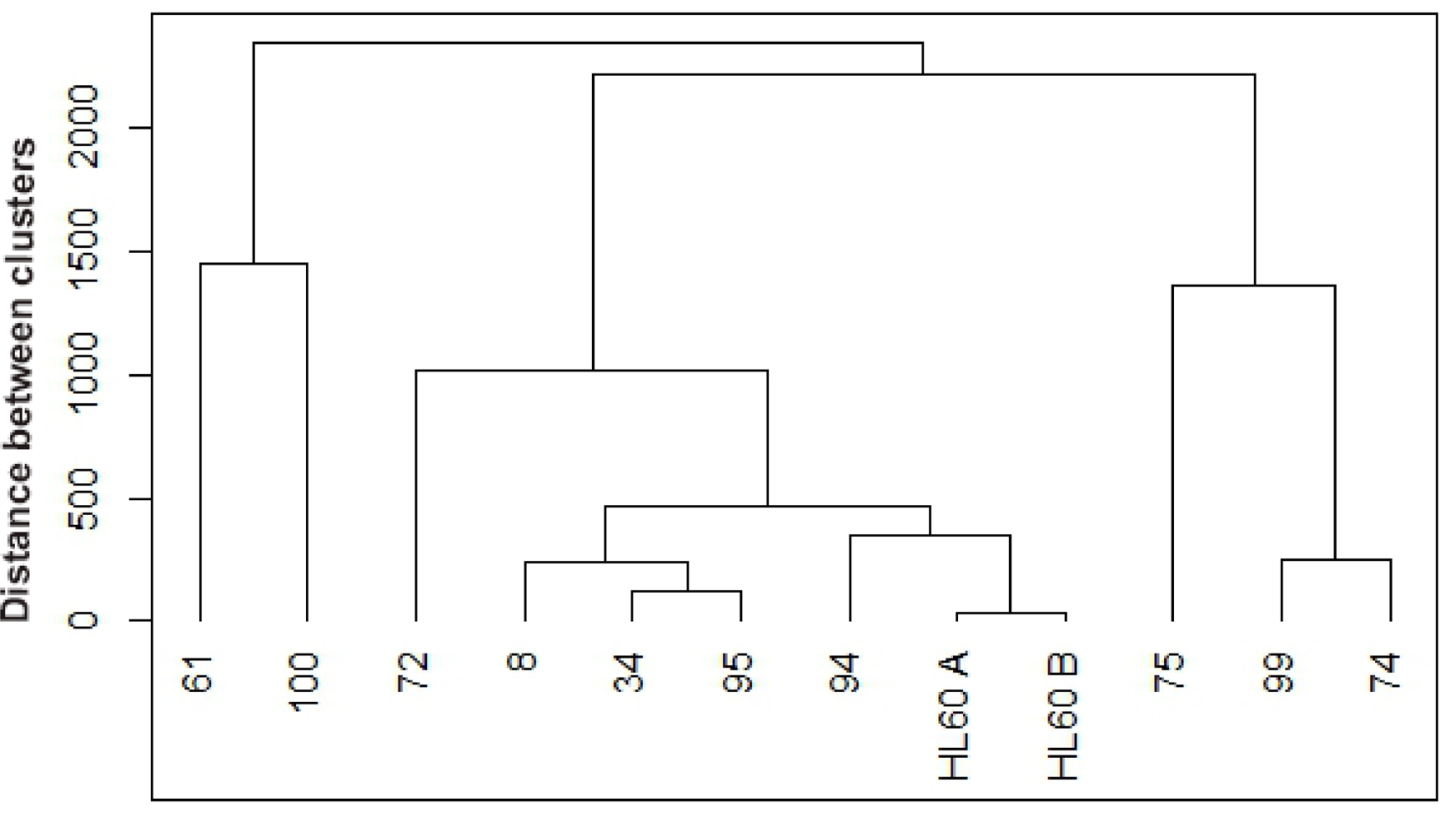
Hierarchical clustering between untreated *de novo* and HL-60 samples based on cluster of differentiation (CD) markers Untreated samples were clustered using R based on CD34, CD38, CD11b, CD14, and CD15 expression. Agglomerative hierarchical clustering analysis was performed using the complete linkage method. *De novo* AML 94 (wt-NPM1) clusters closest with HL-60 cell line (wt-NPM1), the next closer clusters occur at various distances with 7 mut-NPM1samples (AML 8, AML 34, AML 95, AML 72, AML 75, AML 99, AML 74) and then the furthest clusters occur with two wt-NPM1 (AML 61 and AML 100).

Next we added expression of surface differentiation markers to the evaluation. CD34, CD38, CD11b, CD14 and CD15 levels were assessed by flow cytometry. CD34 is a glycoprotein adhesion molecule taken as one of the molecular markers for stemness [48, 49]; CD38 is an ectoenzyme receptor capable of signaling that can drive differentiation [50]; CD11b is a subunit of the integrin receptor, which is also capable of signaling; CD14 is a monocyte differentiation antigen that is as a co-receptor with toll-like receptors for bacterial lipopolysaccharides [51]; and CD15 is a neutrophil carbohydrate adhesion molecule attributed with functions in phagocytosis and chemotaxis and marks Reed Sternberg cells as a telltale for Hodgkin lymphoma [52, 53]. Hence all these cell surface molecules/receptors have attributes associated with signaling and regulation of differentiation. A clustering analysis was performed for untreated samples, based on mean expression of these markers (Figure 3).

Interestingly, when comparing the untreated AMLs, wt-NPM1 AML 94 clusters closely with HL-60 cells (passages designated A and B) and then with several mut-NPM1 AML samples, AML 8, AML 34 and AML 95, and more distantly with the two wt-NPM1 AML 61 and AML 100. It appears that the HL-60 cell line bears fidelity to an AML subtype represented by AML 94.

**Figure 4:**
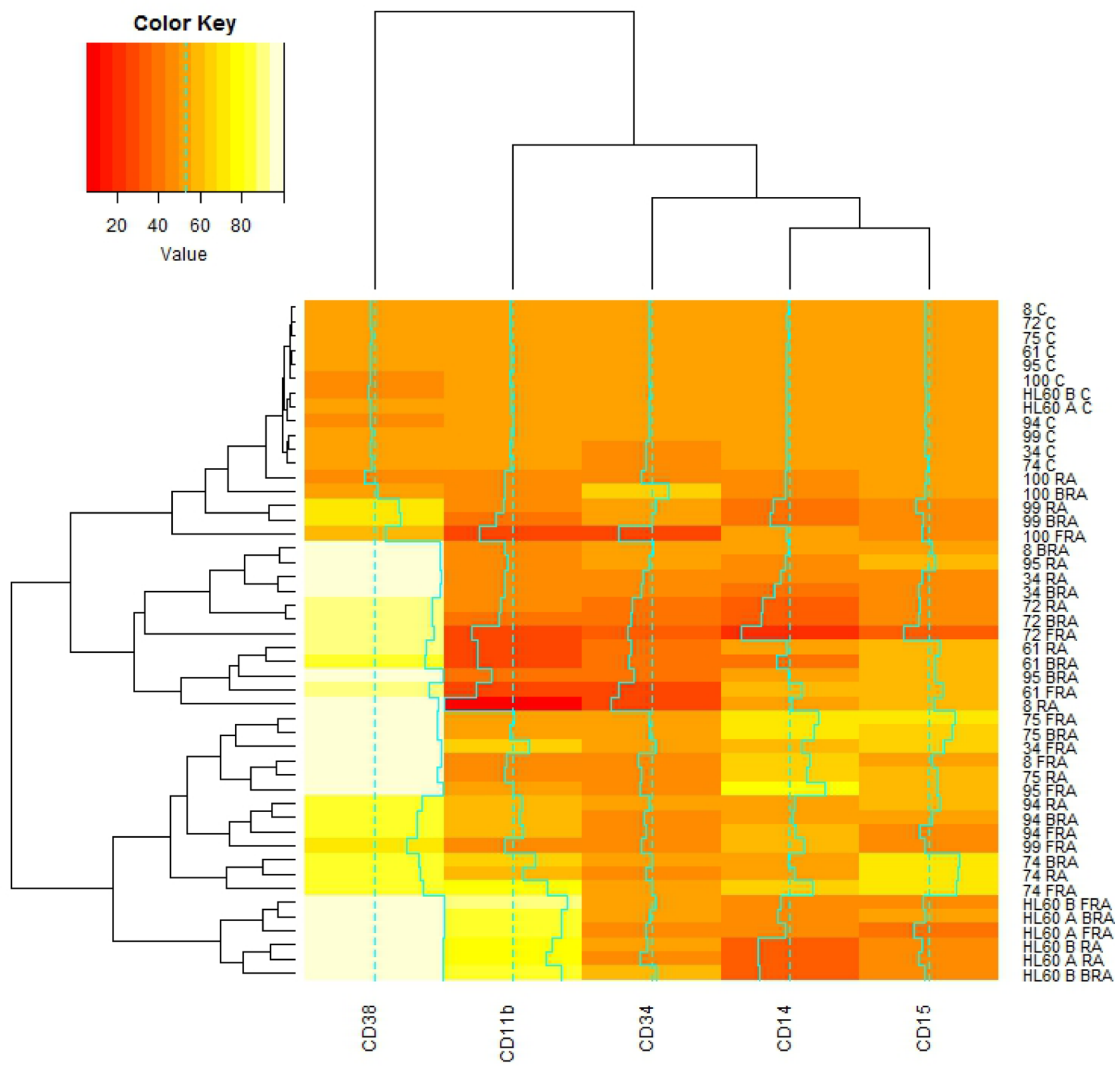
Global correlations of cluster of differentiation markers with samples and treatments *De novo* and HL-60 samples, untreated or RA, FRA, BRA treated for 72 h were assessed for CD34, CD38, CD11b, CD14, CD15 by flow cytometry. The gates were set to 50% of the population positive in untreated samples, and 50% expression is color coded orange. With treatment, the expression of each assessed marker is upregulated (values color coded yellow to white) or downregulated (values color coded with red shades). The correlations of mean expression of those markers for each subject under each treatment were analyzed using the heatmap.2 function available in the gplots package for R.

We next analyzed the percent change with treatment for the same surface markers by flow cytometry, setting gates to exclude about 50% of the untreated cells. We then performed the clustering analysis. We observed that the wt-NPM1 AML 94 cells are again close to the wt-NPM1 HL-60 AML cell line and to the mut-NPM1 AML 99, AML 95, AML 74, and AML 75 cells (Figure 4).

As showed in Figure 4, RA, FRA or BRA increased CD38 in all samples. CD11b, CD14 and CD15 in contrast show a greater variability across different samples for any given treatment. No simple correlation/coupling between these markers and CD34 or NPM1 status was evident. Hence no single marker we measured could be inferred to direct any others or group thereof as in a hierarchical relationship.

**Figure 5:**
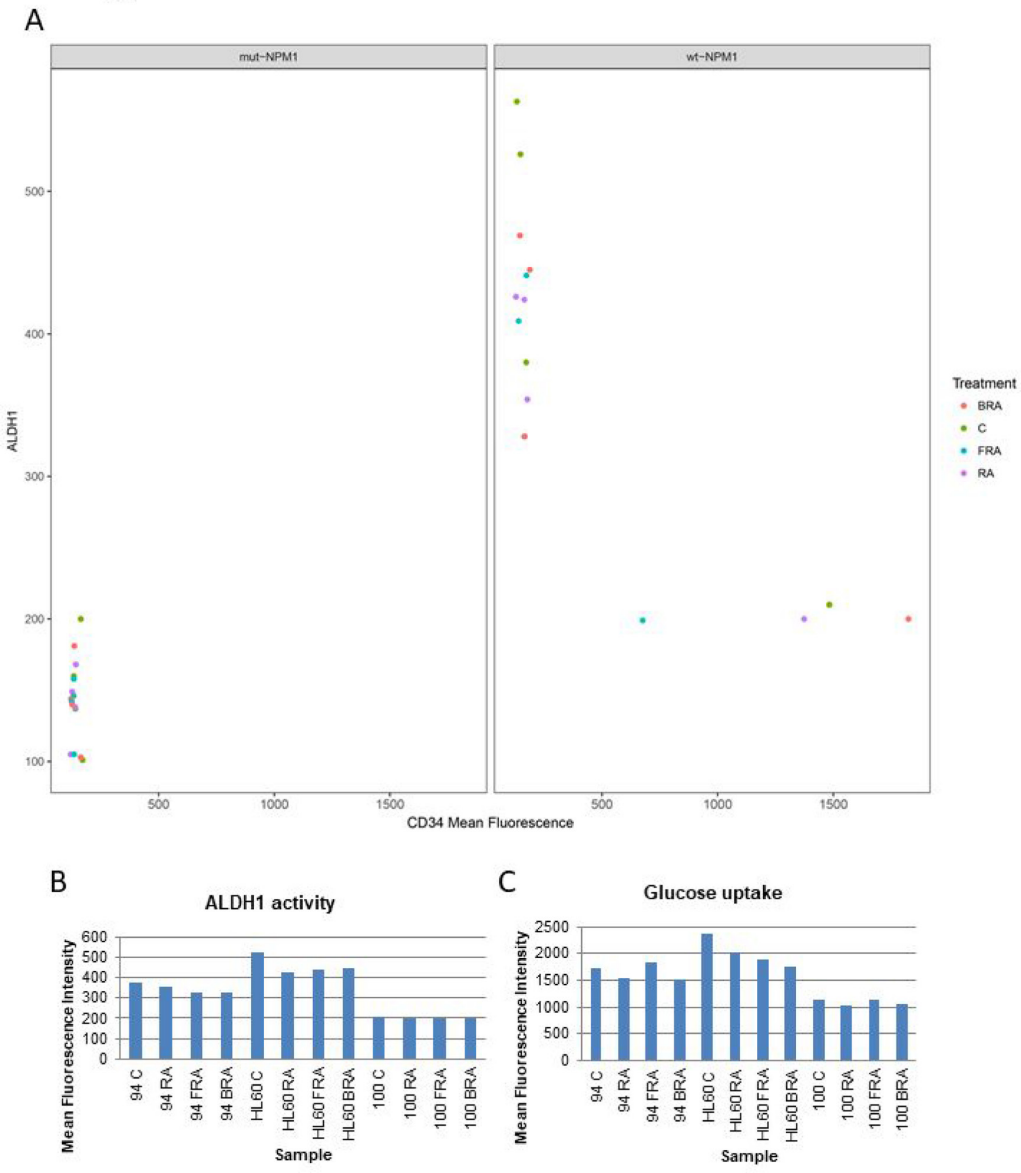
Correlation of CD34 expression, ALDH1 activity and glucose uptake (**A**) Correlation of CD34 expression, ALDH1 activity in mut-NPM1 vs. wt-NPM1. (**B**) ALDH1 activity analyzed using the Aldefluor kit. (**C**) Glucose uptake analyzed by flow cytometry.

ALDH1 has been considered a potential indicator of stemness and poor prognosis. We assessed ALDH1 enzymatic activity in several samples that varied in molecular features described above to explore if any prominent relationships might emerge (Figure 5A and 5B). All the mut-NPM1 samples analyzed have both CD34 and ALDH1 low. However the wt-NPM1 samples have a different expression pattern – one of those parameters tends to be low whereas the other parameter is high (Figure 5A). AML 94 and HL-60 had comparable levels of ALDH1 and CD34 (Figures 2 and 5A). Their response to treatment with RA, FRA or BRA was also roughly similar. Although elevated CD34 and ALDH1 are often coupled and taken as markers of stemness, AML 100, which has high CD34, had low ALDH1. Hence these markers – like the others described above – can uncouple. There is no obvious hierarchical structure. Glucose uptake is a rough reflection of metabolic activity. Here we compared glucose uptake by AML 100, AML 94 and HL-60, representing a gradient of lower to higher ALDH1 and higher to lower CD34 (Figure 5C) and saw that AML 100 had lower glycolytic metabolism than AML 94 and AML 94 had lower glycolytic metabolism than HL-60. The glucose uptake was roughly parallel with ALDH1 and anti-parallel with CD34 in this group chosen to span ALDH1 and CD34 low to high.

### RA alone or in combination decreases number of wt-NPM1 AML cells in colony assays

Although CD34, ALDH1 activity and glucose uptake are often coupled, we identified interesting cases of *de novo* AML samples (AML 94 and AML 100) where they are not coupled. To evaluate this further, we tested the behavior of those two AML cell types, AML 100 with high CD34/low ALDH1/lower glucose and AML 94 with low CD34/high ALDH1/higher glucose, plus, to get an idea of potential dynamic range, an additional two more wt-NPM1 samples, AML 37, AML 76, for colony formation capacity in methylcellulose. We compared cultures that were untreated or treated with RA alone or in combination with either F or B.

**Figure 6:**
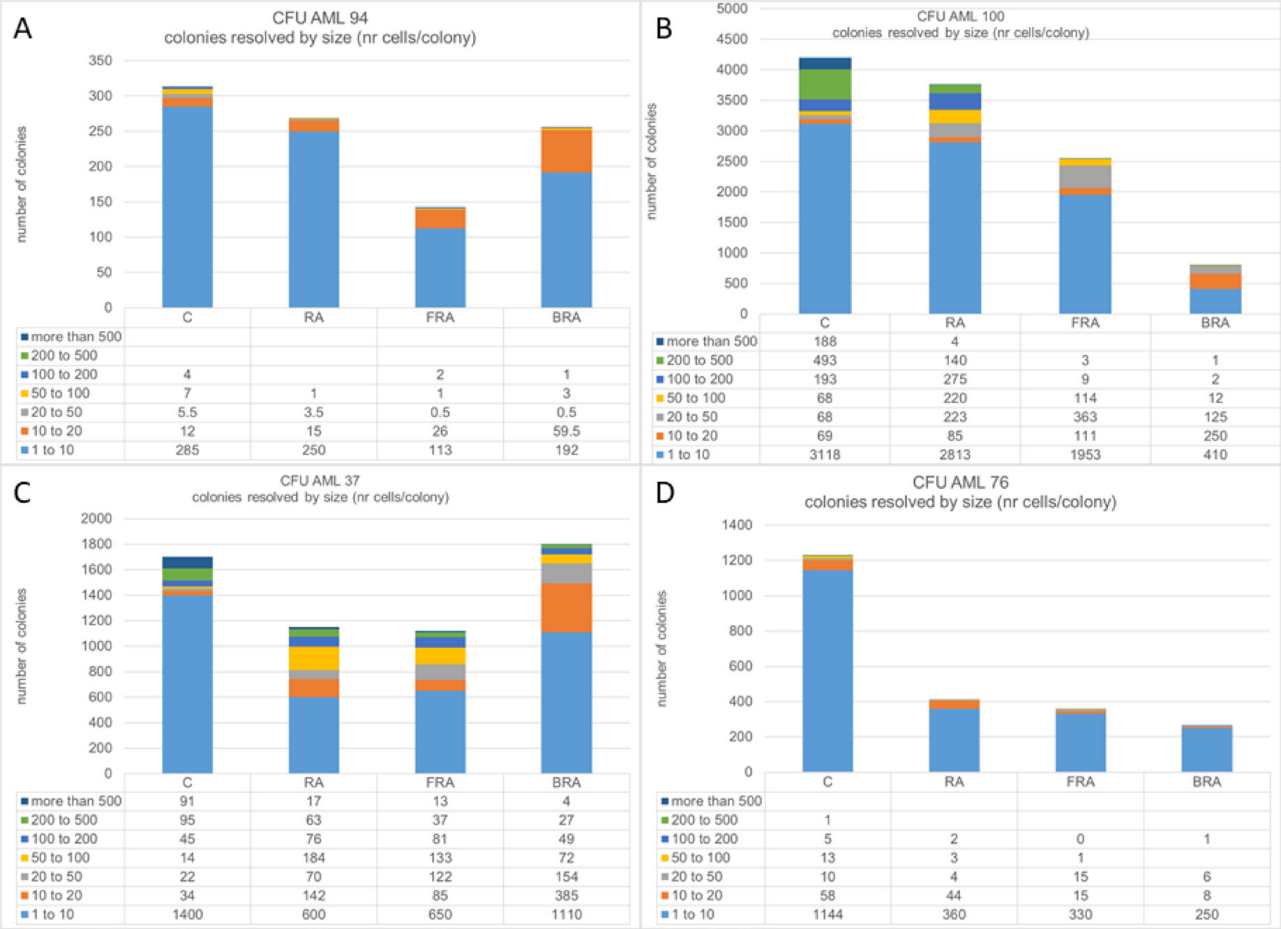
Number of colonies in methylcellulose was assayed for untreated, RA, FRA, or BRA-treated AML 94 (**A**), AML 100 (**B**), AML 37 (**C**) and AML 76 (**D**). For each treatment, the colonies were classified based on number of cells per colony.

The response to RA alone or in combination varied among the four AML samples tested, and in all cases colony size decreased with treatment. (Figure 6). There was great variability in terms of efficacy of RA alone or in combination: AML 94 responded the best to FRA (Figure 6A), AML 100 responded the best to BRA (Figure 6B), AML 37 responded similarly to RA and FRA, but less to BRA (Figure 6C), and AML 76 had the greatest response to RA and the combination added little to the RA response (Figure 6D). It is noteworthy that although all tested samples had some response to treatment, even the best treatment responses in AML 100 and AML 37 still have more cells than the samples from AML 94 and AML 76, although it is not visually evident in the graph because of the large scale needed to include the highest number of cells. When we resolved the total colonies observed for each sample and treatment into cohorts defined by size, we noted that treatment preferentially reduced the number of the largest colonies whereas the small colonies survived better, suggesting that the most proliferatively active cells are more susceptible. Susceptibility was enhanced by combination treatment versus RA alone.

**Figure 7:**
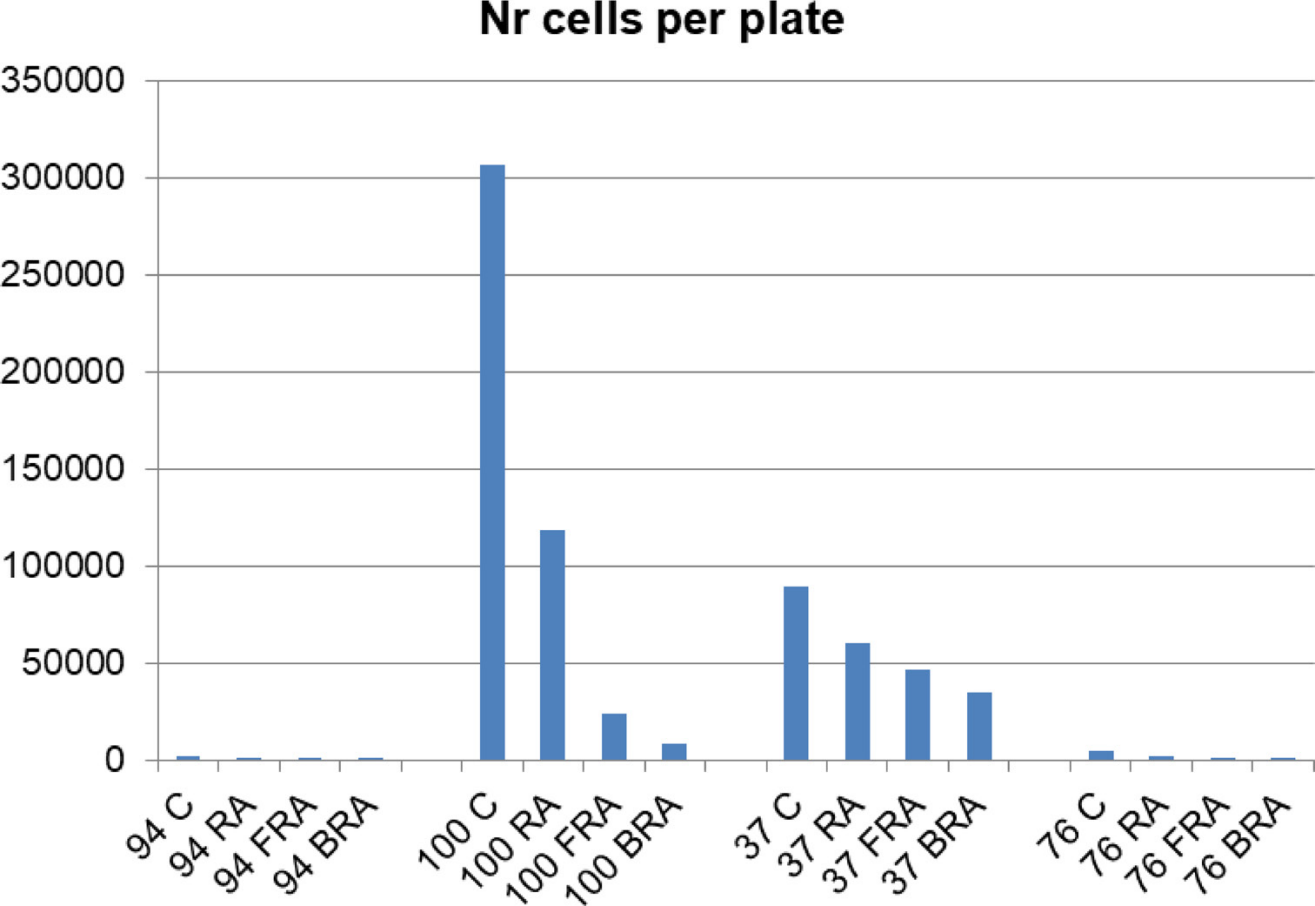
Number of cells per plate was estimated by the sum of the product of the mean number of cells of a size category and number of such colonies for each plate

Since we observed that the treatment decreases the number of large colonies and increases the number of smaller colonies, we next estimated the total number of cells per plate. We calculated this for each plate by adding the numbers obtained by multiplying the numbers of colonies in each category with the mean number of cells in that category. This parameter varies greatly among AML samples (Figure 7), indicating wide dispersion reflecting cells and treatments. AML 94 also had the lowest numbers of cells in methylcellulose, whereas AML 100 had the highest.

### Ability to modulate the c-Cbl/Lyn/c-Raf signalsome is a predictor of decreased colony formation capacity

**Figure 8:**
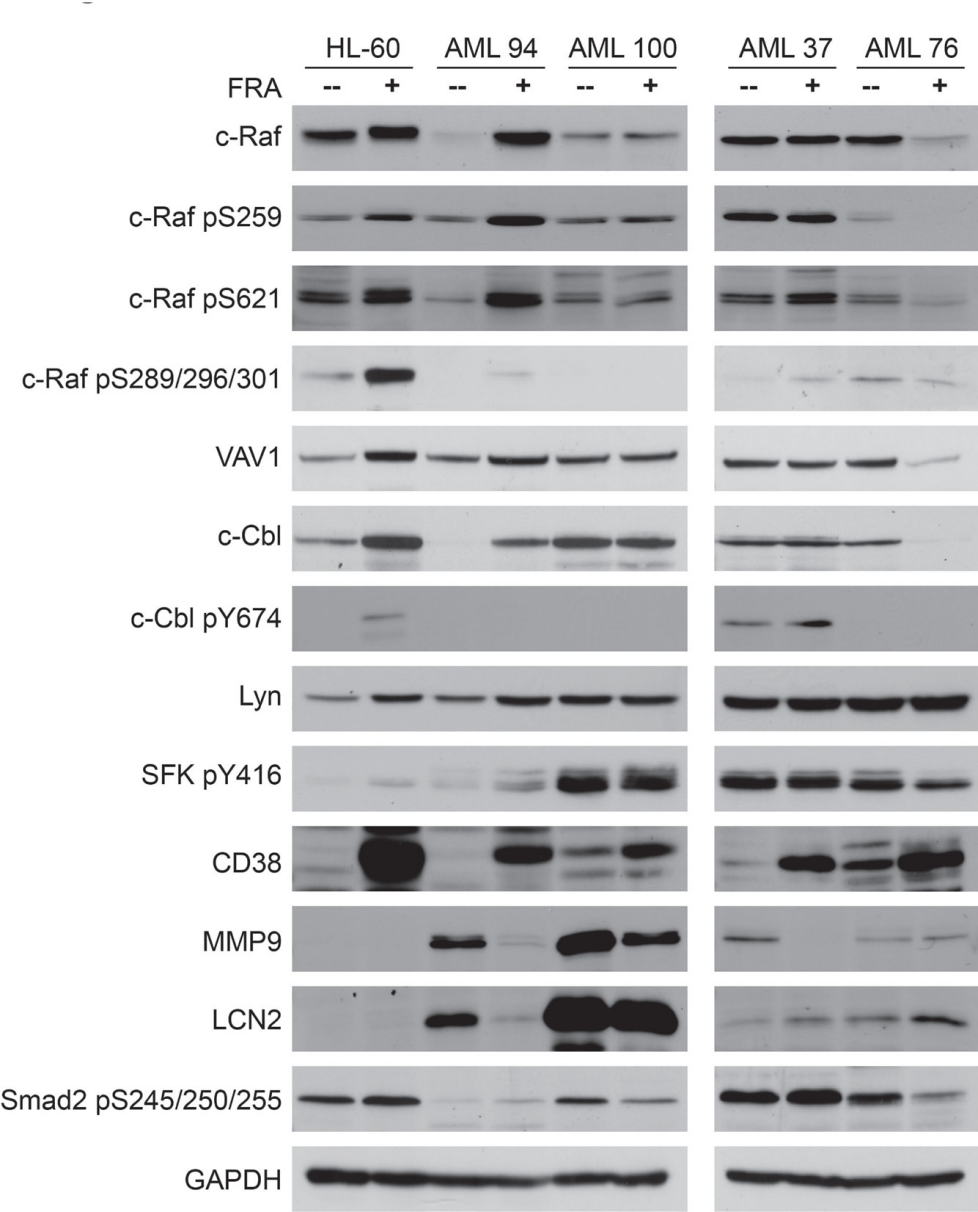
Western blot analysis of 25 µg protein total cell lysate of untreated vs. FRA treated for 72 h samples of HL-60, AML 94, AML 100, AML 37 and AML 76. HL-60 and AML 94 treated with FRA upregulate the expression and activation of most of the signalsome proteins

As FRA was effective in decreasing the number of colonies and the colony size in all samples tested, western blot analysis was performed for several signaling markers for untreated vs. FRA treated samples. Of these c-Cbl [24–26], c-Raf [24, 27–29], Lyn [24, 28], VAV1 [26, 37, 54], and CD38 [26] have been found partnered in a signalsome that generates a signal that regulates proliferation/differentiation and propels RA-induced differentiation of HL-60 AML cells. Smad2 [55], MMP9 [56, 57] and LCN2 [58] have been attributed with roles in AML leukemogenesis. HL-60 was included for comparison to the patient samples, and in particular to determine if it shared any signaling signature similarities with AML 94 (Figure 8).

Since AML 100 and AML 37 had high numbers of cells in methylcellulose whereas AML 94 and AML 76 were in stark contrast with low numbers – indicative of a potentially prominent proliferative difference - we explored their signalsome-related signaling attributes. Figure 8 illustrates that expression and activation of members of the previously reported RA-induced signalsome. c-Raf, VAV1, c-Cbl, Lyn are differentially regulated in AML samples that have high vs. low colony formation capacity. AML 100 and AML 37, which had high numbers of colony forming cells, showed constitutively expressed and activated signalsome components, whereas HL-60, AML 94 and AML 76, although leukemic, retain the ability to modulate the signalsome. In particular it appears that the cells that can regulate c-Cbl in response to treatment have fewer colonies in methylcellulose and those that cannot have more.

In AML 94 and AML 100, MMP9 and LCN2 are highly expressed, but expression decreased with FRA treatment, mirroring decreased number of colonies. Interestingly, in the samples with the highest number of colonies, AML 100 and AML 37, SFK pY416 levels are the highest, and they do not change with treatment. This suite of molecules thus may represent at least elements of a signature for response with prognostic significance.

### c-Cbl/Lyn/c-Raf signalsome defines a subgroup of TARGET AML cohort potentially responsive to RA

**Figure 9:**
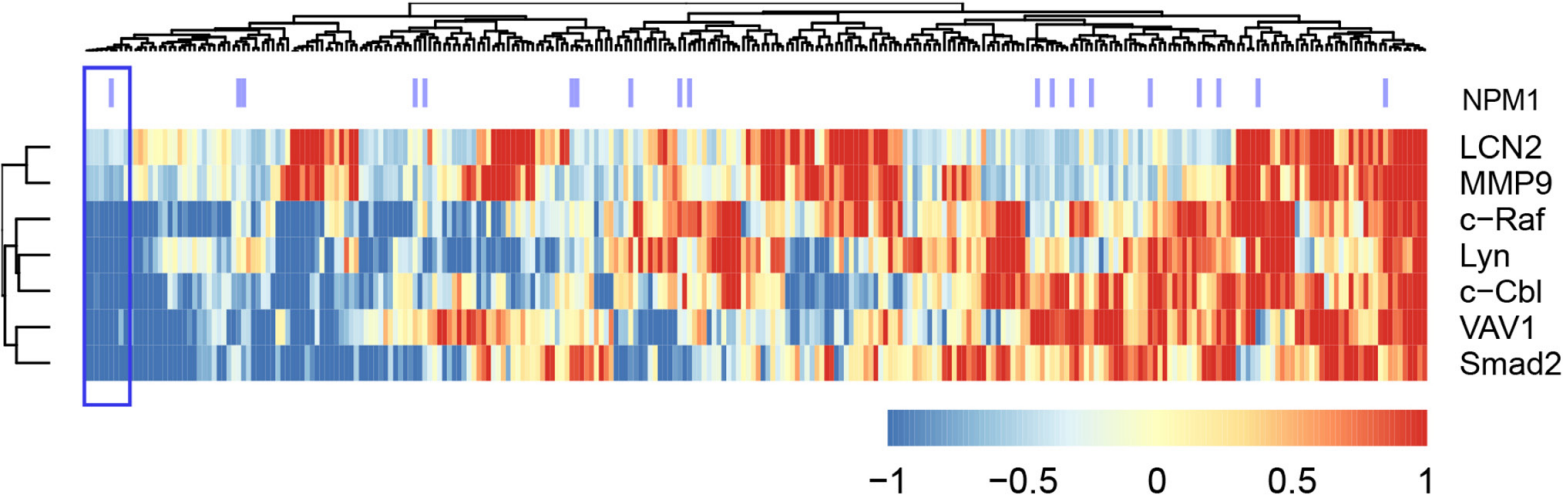
Clustering analysis of the TARGET RNA-Seq data set, using the gene expression data for the genes we analyzed by western blot (Figure 8) Gene expression data points were first z-normalized and then the distance between points was measured using Euclidean distance and then the complete agglomeration method was used in clustering by the ‘hclust’ function in R package ‘stats’. The heatmap using the ‘aheatmap’ function in R package ‘NMF’ (https://github.com/renozao/NMF). Upregulation is color coded red and downregulation is color coded blue.

We next explored whether the ensemble of molecules implicated above betrayed an AML subgroup by a cluster analysis of the TARGET AML RNA-seq data based on the proteins we tested in the 4 wt-NPM1 patients (Figure 9 shows the heat map). Predicated on the AML 94 signalsome signature, we identified a subcluster of patients with such a signature (denoted with purple lines in Figure 9). It ergo appears that there is a subtype of non-APL AML that the HL-60 cell line bears fidelity to and has a signature defined in part by an ensemble of signaling molecules which may be susceptible to differentiation therapy using RA in combination with F or B.

## DISCUSSION

The present studies address the question of whether there is a subtype of non-APL AML that is potentially susceptible to differentiation therapy and if so what molecular signature it might have. We started with an analysis of the public domain TARGET data base for AML RNA-seq. That showed that as previously thought NPM1 wt vs mut provided a stratification showing better DFS for mut-NPM1. We refined that and showed that c-Cbl provided an additional criterion and found that low c-Cbl expression provided a further advantage in DFS. c-Cbl is known to associate with a suite of partners in a signalsome originating signaling that propels RA-induced differentiation and inhibition of proliferation. The expression of elements of this suite was found here to be co-regulated in AML cells. We have previously reported that the signalsome contains a module of interrelated kinases, including Lyn and c-Raf, contributing propulsion to RA-induced differentiation [24, 28, 32, 35]. c-Cbl may have a central regulatory function in the signalsome. c-Cbl is known to regulate SFKs, and we have found that they interact in the signalsome. c-Cbl also interacts with VAV1 in the signalsome. VAV1 has been shown to regulate myelopoiesis, and indeed, a VAV1 KO cripples myelopoiesis [59]. Importantly, VAV1 is a known regulator of the Raf/MEK/ERK MAPK signaling axis, which is imbedded in the signalsome. Significantly, activation of Raf is important for propelling myeloid differentiation. Hence c-Cbl has a prominent potential function governing myeloid differentiation status through such interactions.

Predicated on the emerging significance of the c-Cbl-centered signalsome, we interrogated *de novo* non-APL AML samples that were either untreated or treated with RA alone or in combination with F or B, which are ligands for signalsome AhR and SFK, for cell surface markers that have been used as indexes of differentiation/stemness and have been attributed with signaling capabilities governing proliferation/differentiation. We also assayed colony formation capacity, ALDH1 activity, and glucose uptake, and synthesized this data. It was found that the non-APL AML cell line, HL-60, bore fidelity to a subset of AML. In a set of samples restricted to expressing a range of expression in CD34, ALDH1 and glucose uptake, it was found that colony formation capacity correlated with ability of FRA to regulate a suite of signalsome-related entities, specifically c-Cbl, Lyn, c-Raf/phospho-c-Raf and VAV1. Significantly, the responses due to RA were enhanced by using RA in combination with F or B. Perhaps what is the most interesting is that there is a hitherto undefined subtype of non-APL AML, marked in part by a suit of responsive signalsome molecules that is susceptible to differentiation therapy using RA in combination with other agents that target components of a signalsome seminal to signaling propelling differentiation.

RA differentiation therapy presents a significant reduction in toxicity compared with standard chemotherapy. However it is routinely applicable only in APL and not other cases of AML. Some clinical trials have been motivated by the hope of extending its therapeutic use to other subtypes of AML, targeting in particular mut-NPM1 non-APL AML cases. The mut-NPM1 cases are a minority of non-APL AML. The subtype identified by us and highlighted in Figure 9 is a significant additional group that represents 4% of non-APL AML and includes wt as well as mut-NPM1 patients. A challenge of treating AML is its high heterogeneity; the present report is a significant advance toward resolving some of that heterogeneity to further precision medicine.

## MATERIALS AND METHODS

### Primary sample isolation

Primary samples were acquired in accordance with the Declaration of Helsinki and with Weill Cornell Medicine Institutional Review Board approval.

### NPM1 validation

Total RNA was isolated using the RNeasy extraction kit (Qiagen, Valencia, CA, USA). NPM1 analysis was performed using a massively multiplexed assay that detects all NPM1 insertions in exon 12 at position 863 as previously described [60]. ABL1 was used as an endogenous control and amplified using the standard EAC guidelines and assays described by Gabert *et al.* [61]. Briefly, cDNA was synthesized from 1 μg of total RNA using SuperScript VILO (Life Technologies, Carlsbad, CA, USA). The reactions were carried out at 25°C for 10 minutes, 42°C for 60 minutes and 85°C for 5 minutes. qPCR reactions for NPM1 and ABL1 (as an endogenous control) were performed with Taqman chemistry on a QuantStudio 5K platform (Applied Biosystems, Carlsbad, CA, USA). Annealing temperatures were 58°C for the NPM1 probe set and 60°C for the ABL1 probe set.

### Cell culture and treatments

The primary samples were thawed and cultured as previously described [62]. The Human myeloid leukemia cell line HL-60 was grown as previously described [30]. The cell line was derived from the original isolates, a generous gift of Dr. Robert Gallagher and maintained in this laboratory, certified and tested for mycotoxin by Bio-Synthesis, Lewisville, TX, USA, in August 2017. HL-60 cultures were initiated at a density of 0.1 × 10^6^ cells/mL and primary sample cultures were initiated at 1 × 10^6^ cells/mL. Cell collection for flow cytometry and total cell lysate collection for western blot was done at 72 h post treatment. For CFU assay in methylcellulose, 25000 primary cells were plated in 3 mL culture for 15 days. For treatments, all-*trans* retinoic acid (RA) (Sigma) was added from a 0.5 mM stock solution in ethanol to a final concentration of 1 μM in culture for HL-60 cells and to a final concentration of 150 nM in culture for primary cells, 6-formylindolo(3,2-b) carbazole, i.e. FICZ (F) (Abcam, Cambridge, MA, USA), was added from a 100 μM DMSO stock to a final concentration of 100 nM in culture, bosutinib (B) (Sigma) was added from a 5 mM stock solution in DMSO to a final concentration of 0.25 μM in culture. All reagents were purchased from Sigma unless otherwise indicated.

### Flow cytometry

The primary and HL-60 cells (0.5 × 10^6^) were harvested by centrifugation, and pelleted cells were resuspended in 200 µL of PBS containing 2.5 µL of antibodies against CD11b (clone ICRF44), CD38 (clone HIT2), CD34 (clone 581), CD14 (clone M5E2), CD15 (clone HI98) conjugated either with allophycocyanin or phycoerythrin (BD Biosciences). Following incubation for 1 h at 37°C, cells were analyzed by flow cytometry (LSRII flow cytometer, BD Biosciences). Gates to determine percent change in expression were set to exclude 50% of untreated cells. The mean expression per cell is derived from the entire population gated using FSC and SSC to include all cells and exclude debris.

### Aldehyde dehydrogenase (ALDH1) enzymatic activity

ALDH1 enzymatic activity was measured using the Aldefluor kit (Stem Cell Technologies) by flow cytometry [63–65] as described by Ginestier and coworkers [66], with the modification that the cells were incubated with the substrate at 37°C for 50 minutes instead of 40 minutes [30].

### Glucose uptake

Glucose uptake assay was performed using 6-NBDG (6-(N-(7-Nitrobenz-2-oxa-1,3-diazol-4-yl)amino)-6-Deoxyglucose) [67–69] (Invitrogen) and samples were analyzed by flow cytometry as previously described [30].

### Colony forming unit (CFU) assay

CFU assay was performed as previously described [62]. For each patient sample tested, each treatment was plated in duplicate. The treatments were done at the same concentrations as for the suspension treatments (described in “cell culture and treatments” section).

### Western blot

Total cell lysate (25 µg protein) was resolved by SDS-PAGE analysis [30] using 12% acrylamide gels. Antibodies for western blotting were anti-: c-Raf pS259 (polyclonal), c-Raf pS289/296/301 (polyclonal), VAV1 (polyclonal), Lyn (clone C13F9), SFK pY416 (polyclonal), LCN2 (clone D4M8L), MMP9 (clone D6O3H), Smad2 pS245/250/255 (polyclonal), GAPDH (clone D16H11), HRP anti-mouse, and HRP anti-rabbit (Cell Signaling, MA, USA), c-Raf pS621 (polyclonal) (Thermo Fisher), c-Raf (clone 53) and CD38 (clone 22) (BD Pharmingen, San Jose, CA, USA), c-Cbl pY674 (clone EPR2227) (Abcam, MA, USA) and c-Cbl (polyclonal) (Santa Cruz, CA, USA). All antibodies were diluted 1:1000.

### Data analysis

#### TARGET RNA-Seq data processing

TARGET RNA-Seq data set was downloaded from National Cancer Institute (NCI)’s data portal (https://ocg.cancer.gov/programs/target/data-matrix, May/18/2017). 284 patient samples from the BCCA cohort were used in this study. The processed FPKM (Fragments Per Kilobase of transcript per Million mapped reads) obtained directly from TARGET was converted to TPM (Transcripts Per Million) by exp(log(fpkm) - log(sum(fpkm)) + log(1e6)), and then log2 transformed for further analysis.

### Statistical analysis

All statistics were performed using R (version 3.3.3; http://www.r-project.org/). Survival analysis was used to compare disease free survival between wt-NPM1 and mut-NPM1 groups using the R package ‘survival’ (https://cran.r-project.org/package=survival). A log-rank test was used to compare survival distributions of different subgroups, with *p* < 0.05 considered statistically significant. Gene expression data was z-normalized for clustering analysis using the ‘hclust’ function in R, with Euclidean distance and complete-linkage method. The heatmap was generated using the ‘aheatmap’ function in R package ‘NMF’ (https://github.com/renozao/NMF). The mean expression of differentiation markers for *de novo* samples under each treatment were analyzed using the ‘heatmap.2’ function available in the ‘gplots’ package for R [70].
